# Miscellaneous

**Published:** 1887-08

**Authors:** 


### MISCELLANEOUS.

Steal a Horse.—Abernethy once had for a patient a distinguished gouty nobleman. The nobleman's habits of life were so irregular and indiscreet that Abernethy labored in vain to relieve him of his ailments, and finally one day said to him. "I should advise you, sir, to steal a horse." "What do you mean, Doctor ?" exclaimed the nobleman, in great surprise. "I did not come here to be trifled with." "I am not disposed to trifle with any one, sir. Steal a horse." "Why, Doctor, what do you mean ?" "I mean, sir, if you steal a horse, you will be put where you will have proper food, a proper amount of exercise—where you will be obliged to retire at the proper time for going to bed, and be compelled to rise early in the morning and go to work. In brief, you will be placed where you will be made to lead a rational life ; and this is all that is needed to make a well man of you, sir. Steal a horse."

Filtration of Water.—The efficiency-test of a filter is that it should be able to separate from the water to be filtered bacteria and all kinds of germs, and consequently, all infectious substances which may adhere to them. The distinction between pathogenous and non-pathogenous classes of bacteria, is not of immediate importance in this question, there not being any reason to suppose that a filter which would allow non-pathogenous germs to pass, will destroy or detain infectious matters. On the contrary, the reverse conclusion may be admitted, that a filter which detains all bacteria, would also present a safe protection from infectious matters. In accordance with these views, Flagge investigated most of the usual domestic filters, especially such as spongy iron, coal, stone gravel, cellulose (paper), and found them entirely deficient in the action required of them, and even, under circumstances occasioning a notable increase in the organism, object of filtration. In such cases, the filtered fluid is 100 to 1,000 times richer in germs than the water before the filtration. Experiments with typhus—and cholera—cultures demonstrated that such imperfect filters equally allowed these infectious substances to pass without obstruction.

More favorable results were obtained by variously constructed filters of clay and asbestos, which, at least for a certain time, will furnish water entirely free from germs. Flagge observes that if Hesse pretends having discovered in compressed asbestos and in clay vessels of peculiar density, a filtrating material with permanent purity, such apparatus adapted to practice and domestic use had not been prepared until then. Hesse has stated that filtration pipes of clay, which have filtrated for one to two months free from germs, are permanently germ-tight.—*Tagblatt der* 59 *Naturforscerversammlung zu Berlin.*

Solving a Problem in a Dream.—J. Milton Akers writes as follows: from Pine Island, Minn., to the *Christian Advocate* : "In the winter of 1859–'60 the writer was teaching school in Bedford County, Pennsylvania, and boarding with an intelligent and substantial farmer of German extraction, by the name of Anthony Felton. The family was a remarkable one for ingenuity. One night, after school, the conversation turned upon difficult problems in mathematics. I mentioned one that my brother had sent me which I considered quite intricate. The question was as follows (I reproduce from memory) : 'Sold 5,000 ells Flemish of cloth for $21,250, and gained as much per yard as one-eighth of the prime cost of an ell English. What was the prime cost per yard, and of the whole piece ?' On repeating the question my host told me promptly it could not be done. I repeated it several times for him during the evening, till he had its conditions well fixed in his mind. I assured him I had solved it by algebra, of which he knew nothing. The next morning, on coming from my room, he said : 'I can tell you all about that problem now.' Upon asking him how he had reached the solution, he said : 'I dreamed it out.' I smiled incredulously, for I had no faith in such straight dreaming. I said : 'Let me see your solution ?' and to my astonishment and delight he produced an arithmethical solution that was a marvel of analytic simplicity. I then asked him more particularly about his dream. He said : 'An old man, to whom I had at one time gone to school, came to me in my dream and seeing I was troubled about something, asked the cause. I repeated the question to him and told him that I had told the 'master' that it could not be solved. The old man said to me, 'It makes no difference what you told the 'master,' it can be worked," and then told me how to do it.' And he remembered it so distinctly that he solved the question by the instructions received in his dream.

### A COLLOQUY.

The *tomb* said to the *rose*, sweet flower,
What dost thou with the tears we see
At day break newly watering thee ?

The rose replied, from her leafy bower,
What dost *thou, tomb*, with those we miss
Forever in thy dark abyss ?

For *me*, I make in the shadowy gloom,
Of all those tears a perfume meet
Of amber and of honey, sweet.

O! plaintive flower, replied the tomb
Of every soul that cometh, *I*
Make one more angel of the sky !

—*Translated from the French of Victor Hugo, for* HALL'S JOURNAL OF HEALTH.

